# Nitrogen Assimilation and Transport by *Ex Planta* Nitrogen-Fixing *Bradyrhizobium diazoefficiens* Bacteroids Is Modulated by Oxygen, Bacteroid Density and l-Malate

**DOI:** 10.3390/ijms21207542

**Published:** 2020-10-13

**Authors:** James K. Waters, Thomas P. Mawhinney, David W. Emerich

**Affiliations:** Department of Biochemistry, University of Missouri, Columbia, MO 65211, USA; WatersJ@Missouri.edu (J.K.W.); MawhinneyT@Missouri.edu (T.P.M.)

**Keywords:** nitrogen fixation, *Bradyrhizobium diazoefficiens*, bacteroids, ammonia/ammonium, alanine

## Abstract

Symbiotic nitrogen fixation requires the transfer of fixed organic nitrogen compounds from the symbiotic bacteria to a host plant, yet the chemical nature of the compounds is in question. *Bradyrhizobium diazoefficiens* bacteroids were isolated anaerobically from soybean nodules and assayed at varying densities, varying partial pressures of oxygen, and varying levels of l-malate. Ammonium was released at low bacteroid densities and high partial pressures of oxygen, but was apparently taken up at high bacteroid densities and low partial pressures of oxygen in the presence of l-malate; these later conditions were optimal for amino acid excretion. The ratio of partial pressure of oxygen/bacteroid density of apparent ammonium uptake and of alanine excretion displayed an inverse relationship. Ammonium uptake, alanine and branch chain amino acid release were all dependent on the concentration of l-malate displaying similar K_0.5_ values of 0.5 mM demonstrating concerted regulation. The hyperbolic kinetics of ammonium uptake and amino acid excretion suggests transport via a membrane carrier and also suggested that transport was rate limiting. Glutamate uptake displayed exponential kinetics implying transport via a channel. The chemical nature of the compounds released were dependent upon bacteroid density, partial pressure of oxygen and concentration of l-malate demonstrating an integrated metabolism.

## 1. Introduction

Symbiotic nitrogen fixation is the process whereby rhizobia bacteria enter compatible host plant root cells and form a tumor-like expansion on the root referred to as a nodule [1,2]. The symbiotic bacteria invade plant host cells encased in a plant-derived membrane called the symbiosome membrane, which separates the bacteria from the plant host cell cytoplasm. Within the symbiosome, the bacteria differentiate into nitrogen-fixing forms called bacteroids. The transfer of nitrogen from the bacteroid to the plant is the most fundamental aspect of symbiotic nitrogen fixation, yet much remains to the learned about this process.

Ammonium was shown to be the immediate product of nitrogenase, the enzyme complex that catalyzes the reduction of atmospheric dinitrogen: N_2_ + 8 H^+^ + 8 e^−^ + 16 MgATP → 2 NH_3_ + H_2_ + 16 MgADP + 16 P_i_ [3]. Bacteroids were found to lack the classical glutamine synthetase/glutamine synthase assimilation system and glutamate dehydrogenase, which are the typical pathways by which ammonium is assimilated into organic compounds [4,5,6,7,8,9,10]. Therefore, in the absence of an ammonium assimilation enzyme, it was proposed that gaseous ammonia (NH_3_) diffused across the bacteroid membrane, into the symbiosome space surrounding the bacteroid, was protonated to ammonium (NH_4_^+^), and moved out of the symbiosome by an ammonium transporter in the symbiosome membrane [11,12].

In 1954, Aprison and Burris reported that bacteroids produced amino acids which were released into the incubation medium [13]. Bergersen also reported amino acid release several years later [14]. In 1965, Bergersen incubated detached soybean nodules with ^15^N_2_ and reported that ammonium (NH_4_^+^) was the initial ^15^N-nitrogen-labeled compound produced, but that it was rapidly transformed into other compounds, predominately aliphatic amino acids [15].

In 1978, Meeks et al. employed the short-lived isotope, ^13^N, to determine the immediate products of ammonium assimilation in soybean nodules [16]. Soybean nodules were exposed to ^13^N_2_ for time intervals from 20 s to 15 min. Glutamate and alanine were the prominent compounds labeled at all sampling times except after 20 s of exposure glutamine was the most labeled compound accounting for greater than 80% of total recoverable organic fraction. After 1 min, glutamine declined and glutamate increased to become the organic compound with the greatest amount of labeling. Alanine was labeled beginning at 30 s and continued to accumulate for 15 min.

In 1980, Ohyama and Kumazawa used ^15^N_2_ to identify the ammonium assimilation products in intact soybean nodules exposed for 5 and 10 min [17]. In the nodule cytosol, the greatest labeling of ^15^N was in glutamine followed by glutamate, alanine and allantoin after 10-min exposures. In the bacteroid fraction, ^15^N was rapidly assimilated into alanine followed by glutamate with only minor amounts of glutamine.

In 1985, Kahn et al. proposed a bacteroid nutrient exchange model in which a carbon compound, such as a dicarboxylate, was assimilated by the bacteroid in exchange for a nitrogen compound, such as an amino acid, released to the plant cells [18]. Subsequently, reports appeared indicating amino acids, in addition to or in place of ammonium, were released from bacteroids [19,20,21,22,23,24,25,26,27], but the ammonium diffusion model has remained as the most often cited mechanism of nitrogen transfer.

In 1993, the bacteroid ammonium assimilatory enzyme first proposed by Ellfolk and Katanum [28] was identified as alanine dehydrogenase [29,30]. Smith and Emerich [29,30] reported that *B. diazoefficiens* bacteroid alanine dehydrogenase had a unique kinetic mechanism, a Theorell-Chance mechanism, which has an infinite maximum velocity that could consume all of the nitrogenase produced ammonium [29,30]. Allaway et al. [24] showed in the *Rhizobium leguminosarum*-pea symbiosis that both ammonium and alanine were excreted from bacteroids in assays with a low density of bacteroids and that an alanine dehydrogenase mutant failed to excrete alanine but did excrete ammonium. Plants inoculated with the alanine dehydrogenase mutant had reduced plant biomass. The kinetic mechanism of *R*. *leguminosarum* alanine dehydrogenase is not known. The *R. leguminosarum*- pea symbiosis is an example of an indeterminate symbiosis whereas *B*. *diazoefficiens*-soybean is an example of a determinant symbiosis. In the determinant *Mesorhizobium loti*-*Lotus japonicus* symbiosis, mutation of both alanine dehydrogenase genes resulted in normal plant dry weight [31]. The alanine dehydrogenase of *M. loti* is much less active than that of *B*. *diazoefficiens* and the excretion products from the bacteroids of the double alanine dehydrogenase mutants was not determined. Transcriptomic analysis of the two nodule types show distinct differences in transporter genes. Determinant bacteroid showed an increase in transporters for sugars, taurine and other compounds [32]. Determinant bacteroids also showed an increase in cell surface autoaggregation proteins [32]. Bacteroids are sequestered within symbiosome membranes within nodules. *B*. diazoefficiens bacteroids are sequestered in numbers of up to 10 bacteroids per symbiosome, whereas the number of bacteroids per symbiosome in the *M*. *loti*-*L*. *japonicus* symbiosis is ~1 [33], which is the same as that of the *R*. *leguminosarum*-pea symbiosis [34].

In 2002, Li et al. reassessed the products of nitrogen fixation in soybean root bacteroids using various procedures of isolation and assay and found ammonium to be the main product and had the greatest level of labeling of ^15^N from ^15^N_2_ [35].

The complex environment of the nodule is difficult to replicate *ex planta*, as evidenced by the differing reports on the nature of the molecule(s) bearing atmospheric N_2_ from the bacteroid to the plant. Inherent heterogeneities among studies on *ex planta* bacteroid metabolism are the diverse isolation and assay conditions. Oxygen partial pressure and bacteroid density have been shown to effect *ex planta* bacteroid nitrogen fixation [23,36,37,38]. Most reports on *ex planta* bacteroid metabolism and nitrogen fixation have employed a limited range of conditions of partial pressure of oxygen, bacteroid density or concentration of exogenous metabolites. Here we investigate the effect of the bacteroid density, partial pressure of oxygen and l-malate concentration on changes of ammonia release and uptake and of amino acid transport by *ex planta B*. *diazoefficiens* bacteroids.

## 2. Results

**Figure 1:**
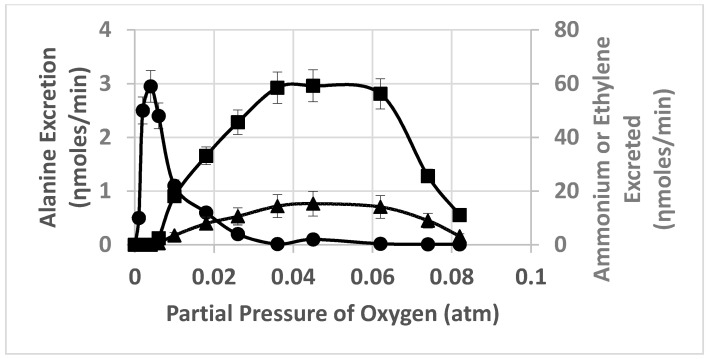
Effect of oxygen partial pressure on alanine excretion, ammonium release and ethylene formation from acetylene by *ex planta B. diazoefficiens* bacteroids. Assays were performed as described in Section 4. Bacteroid amount was 4.8 mg bacteroid dry weight. Values are the mean plus range of duplicate values; circles—alanine, triangles—ammonium, squares—ethylene.

**Figure 2:**
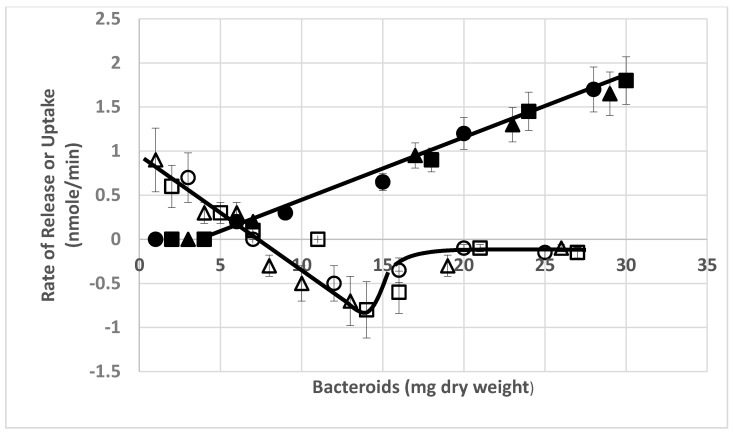
Ammonium release, ammonium uptake and alanine excretion as a function of the amount of *ex planta B. diazoefficiens* bacteroids. Positive values are ammonium release and alanine excretion. Negative values are apparent ammonium uptake. Assays were performed as described in Section 4. Values are the mean plus range of duplicate values. Partial pressure of oxygen was 0.008 atm. Open symbols (circles, squares, triangles)-ammonium; closed symbols-alanine. Each set of symbols represents a separate experiment.

**Figure 3:**
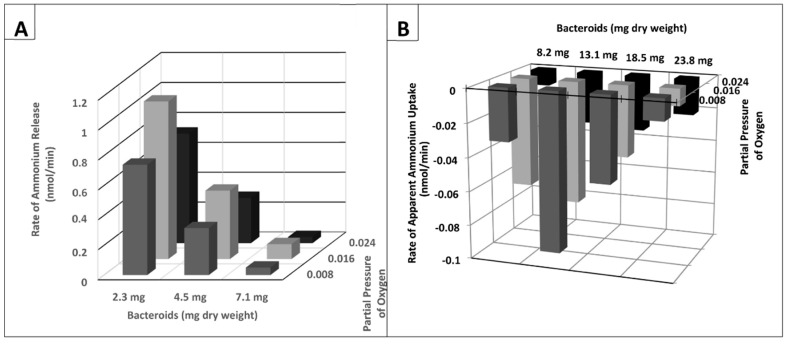
Ammonium release and apparent uptake from *ex planta B*. *diazoefficiens* bacteroids as a function of bacteroid amount and partial pressure of oxygen. (**A**) Ammonium release from bacteroids. (**B**) Apparent ammonium uptake of bacteroids. Note the inverted orientation of Panel B is to emphasize the difference between ammonium release (**A**) and apparent ammonium uptake (**B**) as a function of bacteroid amount. Each column color represents a set of measurements at the same partial pressure of oxygen. Variation of the means among duplicates was ≤12.0%. All assays contained 2 mM l-malate. Each assay was performed in duplicate as described in Section 4. The experiment shown is representative of eight independent experiments each with different ranges of bacteroid densities, partial pressures of oxygen and concentrations of l-malate.

Previous researchers have demonstrated that the partial pressure of oxygen effected the degree of nitrogenase activity of *ex planta* bacteroids as measured by ammonium production, ethylene formed from the alternative substrate, acetylene, or alanine excretion [23,37]. Figure 1 demonstrates the nitrogenase activity as a function of oxygen partial pressure when nitrogen or acetylene are used as substrates. At lower partial pressures of oxygen, nitrogenase produced ammonium was rapidly and efficiently assimilated into alanine, via the novel alanine dehydrogenase, with negligible ammonium released from the bacteroids. This has been documented by^15^N_2_ incorporation into alanine [23]. At greater oxygen partial pressures, ammonium is released from the bacteroids without assimilation to alanine. Acetylene, as an alternative substrate for nitrogenase, is converted to ethylene over the same range of oxygen partial pressures as that observed for ammonium release (Figure 1). However, this result is only observed at higher bacteroid densities in the assay as at lower bacteroid densities alanine release was not detected (Figure 2 and Figure 3). At low bacteroid densities, nitrogenase produced ammonium is the favored product released from bacteroids, but at higher bacteroid densities alanine is the preferred product released (Figure 2 and Figure 3).

**Figure 4:**
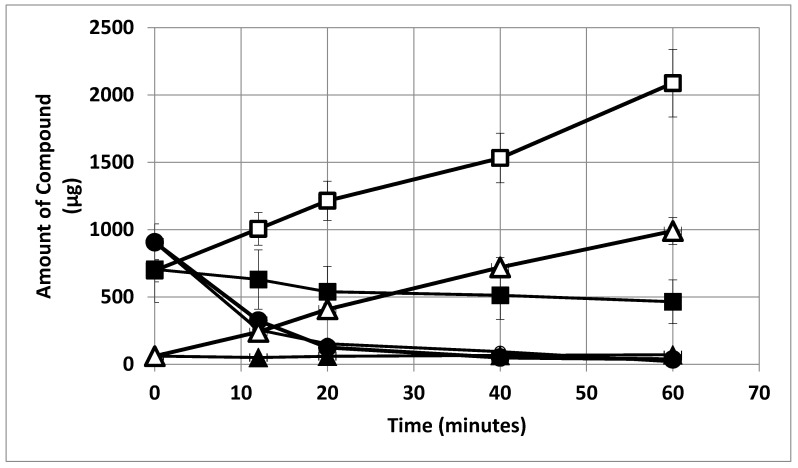
Effect of malate on alanine excretion, ammonium excretion and glutamate uptake by *ex planta B*. *diazoefficiens* bacteroids. Ammonium in the absence of l-malate, open squares; ammonium in the presence of 2 mM l-malate, closed squares; glutamate in the absence or presence of 2 mM l-malate, open and closed circles; alanine in the presence of 2mM l-malate, open triangles; and alanine in the absence of 2 mM l-malate or presence of 2 mM d-malate, closed triangles. Note the symbols for glutamate in the absence or presence of malate overlap. Assays were performed as described in Section 4 and contained 19.1 mg bacteroid dry weight and 0.008 atm partial pressure of oxygen. The values are the means of six measurements plus the range.

Ammonium is apparently taken up at greater bacteroid densities and over a range of partial pressures of oxygen at greater bacteroid densities (Figure 3B). Ammonium uptake by *ex planta B*. *japonicum* (now *B*. *diazoefficiens*) bacteroids has been demonstrated previously by Udvardi and Day [39]. The magnitude of apparent ammonium uptake was small relative to alanine release or glutamate uptake (Figure 4). Ammonium was released from *ex planta* bacteroids in a linear fashion in the absence of l-malate or presence of d-malate. In the presence of l-malate, ammonium levels decline indicating uptake. Alanine release was dependent on the presence of l-malate showing linear accumulation. Glutamate accumulates during anaerobic isolation of the bacteroids, but when exposed to oxygen displays an exponential decline in the absence or presence of l- or d-malate.

**Figure 5:**
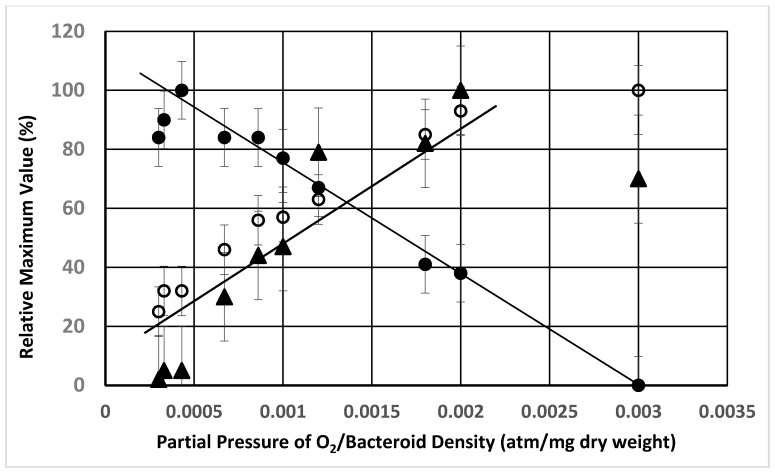
Relative rates of alanine, valine excretion and ammonium uptake from *ex planta B. diazoefficiens* bacteroids compared by the ratio of partial pressure of oxygen divided by the bacteroid density. The maximum rate of excretion at each partial pressure of oxygen (Figure 4) was divided by bacteroid density for alanine (closed circles), valine (open circles) and ammonium (closed triangles). The maximum rate for each nitrogen compound was set at a value of 100% and all remaining rates for that nitrogen compound were determined as a percent of the maximum rate for each nitrogen compound. All assays performed in the presence of 2 mM l-malate as described in Section 4.

The partial pressure of oxygen, bacteroid density and l-malate have profound effects the release and apparent uptake of ammonium and alanine by *ex planta* bacteroids (Figure 1). Alanine and ammonium release can occur simultaneously from *ex planta* bacteroids (Figure 1 and Figure 2) but a plot of the ratio of partial pressure of oxygen/bacteroid density shows that these two nitrogen excretion products react differently (Figure 5). As the ratio of partial pressure of oxygen/bacteroid density increased, ammonium release decreased whereas alanine release increased. Valine, as an example of the branch chain amino acids, which were all very similar, followed a pattern similar to that of ammonium (Figure 5). The transport of these nitrogen compounds is affected by bacteroid density.

**Figure 6:**
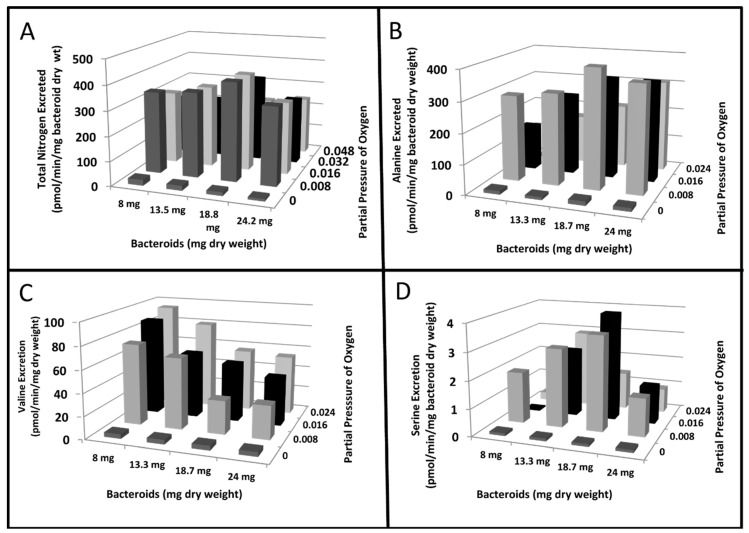
The bacteroid density and oxygen partial pressure landscape of nitrogen compounds transported from *ex planta B*. *diazoefficiens* bacteroids. (**A**) Total nitrogen compounds excreted. Variation of the means among duplicates was ≤18.3%. (**B**) Alanine excreted. Variation of the means among duplicates was ≤14.8%. (**C**) Valine excretion. Variation of the means among duplicates was ≤13.0%. (**D**) Serine excretion. Variation of the means among duplicates was ≤15.3%. Each column color represents a set of measurements at the same partial pressure of oxygen. All assays contained 2 mM l-malate. Each assay was performed in duplicate as described in Section 4. The experiment shown is representative of fourteen independent experiments each with different ranges of bacteroid densities, partial pressures of oxygen and concentrations of l-malate.

A number of amino acids were found to demonstrate linear, oxygen-dependent, l-malate dependent excretion from *ex planta* bacteroids (Figure 6). Each of the represented amino acids demonstrates different conditions for optimal excretion. Figure 6 is presented as rates of excretion and does not consider pre-existing levels of nitrogen compounds that result from the anaerobic isolation. Alanine was the amino acid exported at the greatest levels under many conditions, but the contributions of other amino acids varied considerably. For example, valine was released at a maximum rate of ~100 pmol/min/mg dry weight at 8 mg dry weight of bacteroids and an oxygen partial pressure of 0.024 atm compared to alanine at a maximum rate of ~400 pmol/min/mg dry weight at 18.7 mg dry weight of bacteroids and an oxygen partial pressure of 0.008 atm. Serine was released at a maximum of ~4 pmol/min/mg bacteroid dry weigh at 18.7 mg dry weight of bacteroids and an oxygen partial pressure of 0.024 atm. Leucine and isoleucine were similar to the rates and profile of valine, and threonine was similar to the rates and profile of serine. Averaging over a number of comparable experiments at conditions optimal for alanine excretion (18.7 mg dry weight of bacteroids and an oxygen partial pressure of 0.008 atm), the relative molar amounts of individual amino acids excreted were: Alanine—62%; Valine—8%; Leucine—6%; Isoleucine—4%; Glycine—5%; Threonine—4%; Serine—3%; Phenylalanine—3%; Methionine—2%; Tyrosine—2%; and Lysine—1%. Averaging over a number of comparable experiments at conditions optimal for valine excretion (8 mg dry weight of bacteroids and an oxygen partial pressure of 0.024 atm), the relative molar amounts of individual amino acids excreted were: Alanine—3%; Valine—31%; Leucine—19%; Isoleucine—16%; Glycine—21%; Threonine—0%; Serine—0%; Phenylalanine—1%; Methionine—8%; Tyrosine—0%; and Lysine—1%. Averaging over a number of comparable experiments at conditions optimal for serine excretion (18.7 mg dry weight of bacteroids and an oxygen partial pressure of 0.016 atm), the relative molar amounts of individual amino acids excreted were: Alanine—55%; Valine—9%; Leucine 6%; Isoleucine—4%; Glycine—10%; Threonine—4%; Serine—4%; Phenylalanine—3%; Methionine—2%; Tyrosine—2%; and Lysine—1%. Proline, an osmoprotectant of bradyrhizobia [40] was present in the bacteroid suspensions at levels usually ~1%, and never varied appreciably over time in the absence or presence of l-malate. Histidine, aspartic acid, arginine, cysteine, glutamine, asparagine and tryptophan were rarely found in the bacteroid suspensions appearing transiently at low levels.

**Figure 7:**
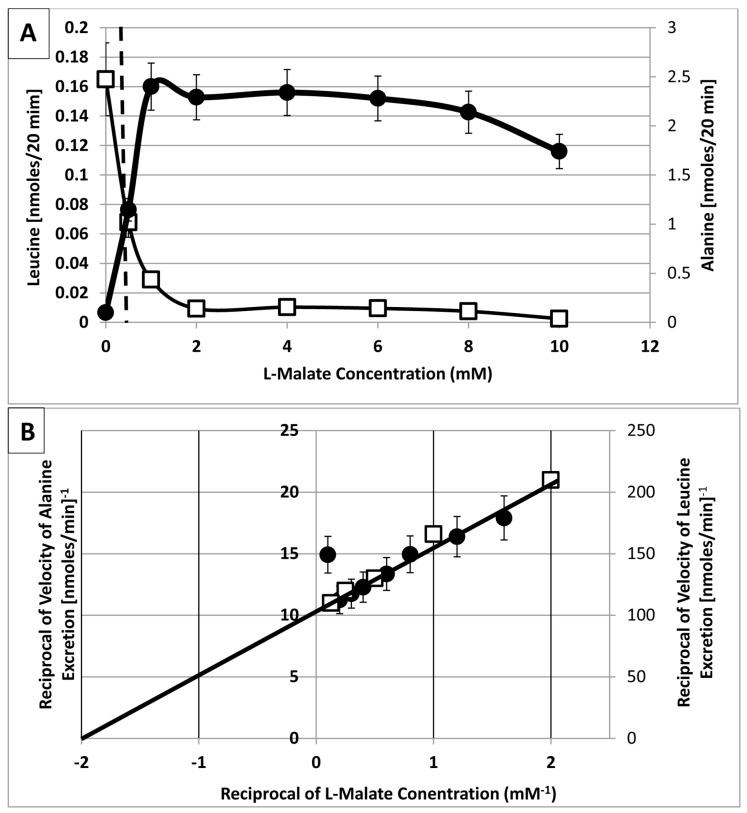
Alanine and leucine transport by *ex planta B*. *diazoefficiens* bacteroids. (**A**) Alanine and leucine transport from bacteroids as a function of malate concentration. The dashed line represents 0.5 mM l-malate. (**B**) Lineweaver-Burk plot of the reciprocal rate of alanine and leucine excretion versus the reciprocal of l-malate concentration. Absolute values of the leucine concentrations were used from (**A**) for inclusion in (**B**). Assays performed as described in Section 4 containing 18.4 mg dry weight of bacteroids and 0.008 atm partial pressure of oxygen. Each value is the mean of duplicates plus the range. The experiment shown is representative of two separate experiments. Alanine, closed circles; leucine, open squares.

The organic acids, malate, succinate and fumarate have been known to support the greatest rates of *ex planta* nitrogen fixation of bacteroids [25,37]. Malate was the most abundant citric acid cycle intermediate in soybean nodules [41]. Alanine excretion was dependent on the presence of malate and oxygen partial pressure (Figure 4). Alanine excretion was dependent upon the malate concentration yielding hyperbolic kinetics allowing for the estimation of a K_0.5_ value for alanine excretion of~0.5 mM l-malate (Figure 7). High concentrations of malate ≥ 10 mM appeared to inhibit alanine excretion. In contrast to alanine excretion, which was dependent upon the concentration of l-malate, the excretion of leucine (Figure 7), valine, isoleucine, glycine, methionine, serine and threonine excretion were inhibited by l-malate, but not d-malate, yielding a K_0.5_ value of ~0.5 mM l-malate. The hyperbolic results, typical of Michaelis-Menten kinetics, suggest a single rate-defining process, such as an enzymatic reaction or a membrane transport process such as l-malate uptake or alanine release [42].

**Figure 8:**
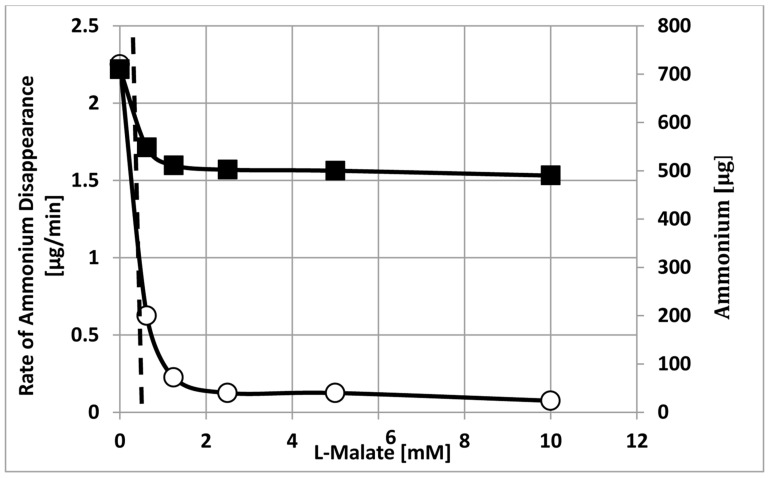
Rate of ammonium uptake and ammonium levels of *ex planta B*. *diazoefficiens* bacteroids in the presence of l-malate. The dashed line indicates a K_0.5_ value of ~0.5 mM l-malate. Assays were performed as described in Section 4 and contained 18.7 mg bacteroid dry weight and 0.008 atm partial pressure of oxygen in the presence of varying amounts of l-malate. Amount of ammonium, closed squares; rate of ammonium uptake, open circles.

As reported by others [36,43], ammonium was released from bacteroids during their anaerobic isolation from nodules at seen in Figure 4 at the beginning of the assay. Ammonium continued to be released at low bacteroid densities and higher partial pressures of oxygen in the absence of l-malate or presence of d-malate (Figure 2 and Figure 3). The presence of l-malate at high bacteroid densities and low partial pressures of oxygen suppressed ammonium release and ammonium disappeared from the external assay volume as a hyperbolic function with a K_0.5_ value of ~0.5 mM malate (Figure 8). The rate of ammonium disappearance also yielded the same K_0.5_ value of ~0.5 mM l-malate. The simple hyperbolic kinetics is indicative of an enzyme-catalyze reaction or a membrane transport process [42].

**Figure 9:**
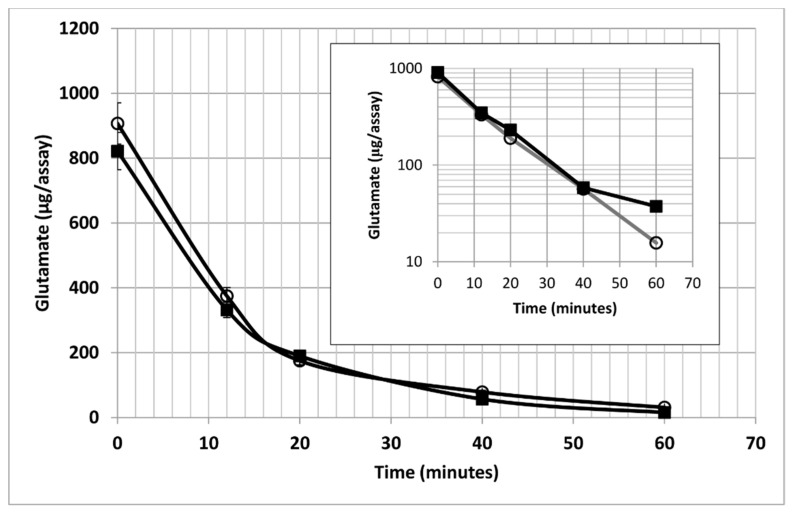
Glutamate uptake by *ex planta B*. *diazoefficiens* bacteroids in the absence and presence of 2 mM l-malate. The insert is a semi-log plot of the same data in the main figure. Assays performed as described in Section 4 containing 14.1 mg dry weight of bacteroids and 0.016 atm partial pressure of oxygen. Absence of l-malate, closed squares; presence of l-malate, open circles. The values are the means of six measurements plus the standard deviation.

In the absence of oxygen, glutamate was released by bacteroids (Figure 9). In the presence of oxygen, glutamate levels outside of bacteroids declined in an exponential manner independent of l- or d-malate indicating the glutamate uptake kinetics were not influenced by malate-related metabolism, such as the citric acid cycle (Figure 4). Exponential kinetics are indicative of an open ion channel [44,45].

No significant differences were found between sucrose density purified bacteroids and Percoll purified bacteroids. The results presented here were from sucrose density purified bacteroids.

## 3. Discussion

**Figure 10:**
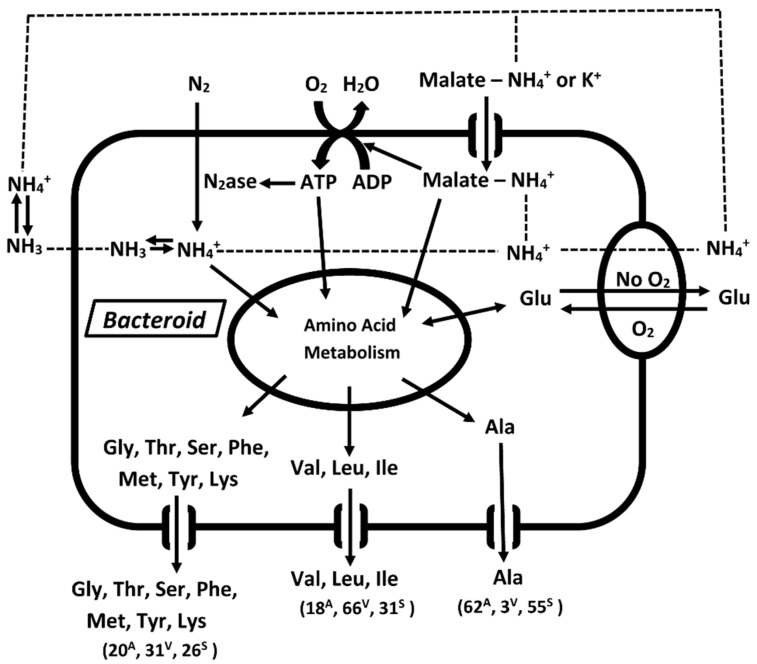
Model of *ex planta B*. *diazoefficiens* bacteroid nitrogen compound transport. The three sets of amino acid transporters do not represent specific transporters but rather groupings of amino acids to demonstrate the variations in amounts of amino acids released under three sets of conditions: conditions for optimal alanine excretion, optimal valine excretion and optimal serine excretion (Figure 6). The values in parenthesis are the molar% of indicated compound(s) relative to the total of all amino acids released under each condition. The superscripts indicate the optimal conditions for alanine (A), valine (V) and serine (S). Individual values for each amino acid are given in the text describing Figure 6. Ammonia may diffuse from the bacteroid and ammonium taken up via other transporters (dotted lines).

A mixture of nitrogen compounds was found to be released from *ex planta B*. *diazoefficiens* bacteroids as the experimental conditions changed (Figure 6 and Figure 10). The excretion of individual amino acids varied greatly over the landscapes (Figure 6B–D), but the sum total of all released amino acids resulted in fairly uniform totals across all conditions (Figure 6A). This demonstrates that *ex planta* bacteroids have the ability to adapt their amino acid metabolism and transport to the experimental conditions to maintain a continuous supply of amino acids in the assays. Bacteroid density, the partial pressure of oxygen and l-malate were found to affect the nitrogen assimilation products released from *ex planta B. diazoefficiens* bacteroids. The partial pressure of oxygen has an obvious effect as it is needed to produce ATP for nitrogenase, the oxygen-labile enzyme that reduces atmospheric dinitrogen. Bacteroid density obviously affects the oxygen availability of each bacteroid within the *ex planta* assay, but it also may be a parameter that adjusts the optimal concentrations of inter-cellular signal transduction compounds among the *ex planta* bacteroid population because of the absence of the symbiosome space and membrane. The symbiosome membrane of soybean nodules encompass up to 10 bacteroids. Bacteroids amount to 40% of soybean nodules by weight and up to 20,000 bacteroids occupy ≥75% of the total volume of each infected cell [1]. Determinant bacteroids, such as *B. diazoefficiens*, had greater levels of expression of autoaggregation proteins [32], which would affect the cellular interactions as a function of bacteroid density. Bacteroid aggregation may be similar to a biofilm. Biofilms are a syntrophic consortium of microorganisms adhering to surfaces that exchange signals to synchronize metabolic functioning of the entire bacterial population [46]. In legume nodules, changes in the diffusion of oxygen occurs over the course of a few minutes by existing physiological mechanisms [47,48,49]. These rapid changes in response to oxygen partial pressure may alter the nitrogen assimilation products. While the number of bacteroids per nodule cannot change, the functional population may change via metabolic regulation perhaps related to those metabolites/signal molecules that regulate oxygen diffusion [47,48,49].

Glutamate and ammonium are known modulators of metabolism in bacteria and bacterial biofilms [46,50,51] and glutamate has been shown to be a signal molecule in plants [52,53]. Glutamate and ammonium were both taken up and released by bacteroids under different conditions making them potential bio-indicators [19,54,55,56,57]. Ammonium uptake by bacteroids has not been demonstrated *in planta*. Based on the kinetics of their removal from the external medium, glutamate appeared to be transported via an ion channel (Figure 9) and ammonium appeared to be taken up by a membrane transporter (Figure 8). The kinetics are noteworthy as exogenous malate affected nitrogen metabolite release or uptake. Such a direct kinetic relationship can occur only if transport, not metabolism, is the rate limiting process.

Prindle et al. have shown that ion channels provide a mechanism for signaling in cellular communities as they allow for rapid movement for effective signal transduction [46]. Liu et al. reported oscillatory dynamics between peripheral and interior cells of a biofilm of *Bacillus subtilis* in which the peripheral and interior cells compete for glutamate but share ammonium [50]. Bacteroids exhibited similar behavior as glutamate was rapidly taken up in the presence of oxygen leaving low levels detectable outside the cells (Figure 4 and Figure 9). In contrast, ammonium appeared to reach equilibrium, thus allowing it to be shared among the bacteroids (Figure 4 and Figure 8). Glutamate does not cross the symbiosome membrane of soybean [58] limiting its role to inter-symbiosome functions. Tyerman et al. reported that the symbiosome membrane has an ammonium channel [59], which means ammonium could serve as an intra-cellular communicator as well as a substrate for assimilation within the plant. When the level of glutamate outside of the bacteroids reached a minimum, ammonium remained unchanged, or reached equilibrium, and alanine excretion was maximized. Adjustment of bacteroid density may not merely adjust oxygen concentration, but also provide the *ex planta* bacteroids with the appropriate concentrations of extracellular metabolites necessary for optimum bacteroid functioning. Bacteroid autoaggregation proteins would promote intercellular signaling.

With few exceptions [24], only a single nitrogen compound has been considered to be transported from bacteroids. Figure 6 shows that a mixture of amino acids was released at each combination of oxygen partial pressure and bacteroid density. The inverse nature of the partial pressure of oxygen/bacteroid density interaction (Figure 5) shows that alanine responds differently than ammonium or the branch chain amino acids. *Ex planta* bacteroids are highly adaptable to changes in experimental conditions responding by changing the chemical forms of the nitrogen compounds released. Comparing Figure 6, Panel A to the other panels shows that the changes in total nitrogen compounds changes comparatively less and more moderately than the individual compounds represented in this figure (Panels B–D). This demonstrates an ability, by *ex planta* bacteroids, to transport a uniform amount of nitrogen compounds over a variety of experimental conditions. This result may explain why there is little effect on plant dry weight when inoculated with alanine dehydrogenase mutants [24,31]. The symbiotic system adjusts by providing alternative nitrogen compounds when one is limited.

Streeter found an extremely low concentration of ammonium in the plant nodule cytosol [60]. The pH optimum of bacteroid enzymes has been reported between pH 7.9 and 8.6 [29,30,61,62,63,64,65,66]. Based on the pKa of ammonia/ammonium of pH 9.2, at a pH of ~8 within the bacteroid, approximately 10% of the ammonia/ammonium pool would be gaseous ammonia. At pH 6.5, the ammonia/ammonium ratio would be ~0.2%. The pH compartmentation would facilitate the diffusion of gaseous ammonia out of the bacteroid to the symbiosome space or plant nodule cytosol where it would be ionized to ammonium. As a physical-chemical process, ammonia diffusion out of the bacteroid should continue in the presence of l-malate, but the external ammonium concentration decreased in a hyperbolic fashion with increasing l-malate with a K_0.5_ of 0.5 mM (Figure 8). This indicates that in the presence of l-malate, ammonium must be taken up by the bacteroids. The l-malate effect on ammonium release could be explained by ammonium co-transport with l-malate uptake via the dicarboxylate transport system as it requires a monovalent cation [67,68].

Alternatively, Howitt and Udvardi [69] proposed that potassium channels and water channels may also provide a route for the transport of ammonium. The hydration shell of water surrounding ammonium is similar to that of potassium and only ~10% smaller than the hydration shell of sodium [69] indicating ammonium could serve as a substitute for potassium or sodium ions. Potassium is needed for nodule formation, but once nodules are formed nitrogen assimilation was not affected by adding potassium to nodulated plants [70,71]. The use of ammonium as a counter-ion in place of potassium would reduce the potassium requirement and potentially alleviate the decline in nitrogen fixation capacity in potassium deficient soils. Many enzymes can utilize potassium, sodium and ammonium equivalently to maintain cellular functions [72,73]. Hoelzle and Streeter demonstrated that ammonium served as a better ion for rhizobium trehalase activity than potassium [74].

Buurman et al. reported that ammonium could replace potassium ions in chemostats of potassium-limited *Escherichia coli* [75], *Klebsiella pneumoniae* [75]) and *Bacillus stearothermophilus* [76]. Ammonium ions were taken up via the high affinity potassium uptake system. The uptake of ammonium ions coupled to the diffusion of ammonia gas out of the cells resulted in an ammonia/ammonium cycle that was coupled to the extrusion of protons to maintain ionic balance across the bacterial membrane and promote energy generation [76]. An ammonia/ammonium cycle has also been observed in animal cells [77] suggesting it is a common biological process. An ammonia/ammonium cycle of *B*. *diazoefficiens* bacteroids coupled to the net uptake of l-malate would display transporter-type kinetics (Figure 8). The cycle would be uncoupled in the absence of l-malate and would result in the net release of ammonia from the bacteroids (Figure 4). In the presence of l-malate, the ammonium levels remained fairly constant (Figure 4) suggesting the operation of an ammonia/ammonium cycle (Figure 10) which could be integrated into amino acid transport, dicarboxylate uptake, energy generation, ionic balance and signal transduction. Ammonium via an ammonia/ammonium cycle may act as a physiological buffer to communicate changes of environmental conditions to adjust bacteroid metabolism. An ammonia/ammonium cycle would not be established in a flow-through assay system [33], which removes regulatory/communication compounds resulting in net ammonium export.

The commonality of several processes related by a similar K_0.5_ value for l-malate indicates that it was not merely a substrate for bacteroid energy generation, but regulates and integrates bacteroid nitrogen metabolism and transport. Symbiotic nitrogen fixation is an overwhelming sink for photosynthetic compounds to the extent that it may limit overall plant yield [78,79,80,81]. l-malate, a product of photosynthetically-derived carbon, acts as an indicator of photosynthetic capacity, the concentration of which in turn was determined by environmental parameters. Although l-malate concentration changed the amino acid and ammonium composition in the assay solution, the resiliency of the symbiotic system would provide a fairly constant total nitrogen content to the plant under a wide range of conditions (Figure 6).

## 4. Materials and Methods

Soybean (*Glycine max* L. cv Williams 82; Foundation Seed, University of Missouri, Columbia, MO, USA) were inoculated with *Bradyrhizobium diazoefficiens* and grown as described [82,83]. Percoll purified bacteroids were prepared as described by Allaway et al. [24]. Sucrose density gradient purified bacteroids were prepared as described by Waters et al. [61] and resuspended in 50 mM tricine, 5 mM potassium phosphate, 0.5 mM MgCl_2_ and 0.1 mM EDTA, pH 8.0 (TMEP) after washing once in the same buffer. One mL of TMEP assay buffer with or without 0.625 to 12 mM d- or l-malate was added to a 14 mL vial, sealed with a serum stopper and evacuated and refilled with purified N_2_ four times. Oxygen was added to each vial via a glass syringe to the desired partial pressure, 0.005 to 0.030 atmospheres. Bacteroids equivalent to undiluted OD_600_ of 10 to 60 were added to the vials to start the assay and shaken at ~80 rpm at room temperature. Each experimental condition was performed in duplicate or triplicate. At timed intervals or 8, 10 or 12 min for a total time of 40, 50 or 60 min respectively, the assay vials were opened and the contents poured into microfuge tubes and immediately centrifuged at 11,000× *g* for 20 s. The clear supernatant was removed quickly and frozen in dry ice. They were stored at −20 °C until analyzed for amino acids and ammonium by cation-exchange chromatography coupled with post-column ninhydrin derivatization and quantitation on L8900 Hitachi Amino Acid Analyzers (Hitachi High-Tech America, Inc., Schaumburg, IL, USA; Hitachi-hta.com). Compounds measured include all 20 protein-containing common amino acids, ornithine, taurine, hydroxyproline, hydroxylysine, and ammonium. Norleucine and amino caproic acid were used as internal standards. The results are plotted as mg dry weight as there was some variability in the fractions apparently due to compounds absorbing at 600 nm. The data were more comparable when converted to mg dry weight of bacteroids. Acetylene reduction was performed in the assay vials with the addition of 1.4 mL of acetylene. Gas samples (0.2 mL) were removed via syringe at four-minute intervals over 20 min and injected into a Varian 600D gas chromatograph equipped with a flame ionization detector (Varian Medical Systems, Palo Alto, CA, USA; Varian.com) and a Porapak N column (Waters Corporation, Milford, MA, USA; Waters.com) Each experiment was performed in duplicate or triplicate. Rates of nitrogen compound transport and acetylene reduction were determined by linear regression analysis using the least squares method. Buffers, chemicals and reagents were obtained from Millipore Sigma (St. Louis, MO, USA. Sigmaaldrich.com)

## 5. Conclusions

*Ex planta* bacteroids are highly adaptable in responding to various experimental conditions by transporting differing mixtures of nitrogen compounds into the assay medium, but in sum total resulting in a fairly uniform amount released under a breadth of conditions. This implies the bacteroids have a metabolic plasticity to alter the chemical nature of nitrogen compounds to the plant as environmental conditions change. Dependence on a single nitrogen compound or a small group of nitrogen compounds may limit symbiotic efficiency, and by implication, plant growth and development, as environmental conditions change. The similar K_0.5_ for l-malate for alanine, branched chain amino acids and ammonium exchange demonstrates a coordinated integration of bacteroid nitrogen metabolism and transport, and that transport may be rate-limiting.
